# Declined expressions of vast mitochondria-related genes represented by CYCS and transcription factor ESRRA in skeletal muscle aging

**DOI:** 10.1080/21655979.2021.1948951

**Published:** 2021-07-06

**Authors:** Jingbao Kan, Yifang Hu, Yaoqi Ge, WenSong Zhang, Shan Lu, Cuiping Zhao, Rihua Zhang, Yun Liu

**Affiliations:** a Department of Geriatrics, The First Affiliated Hospital of Nanjing Medical University, Nanjing, China; b Department of Medical Informatics, School of Biomedical Engineering and Informatics, Nanjing Medical University, Nanjing, China

**Keywords:** Skeletal muscle aging, mitochondria, gene expression, CYCS, ESRRA

## Abstract

Age-related skeletal muscle deterioration (sarcopenia) has a significant effect on the elderly’s health and quality of life, but the molecular and gene regulatory mechanisms remain largely unknown. It is necessary to identify the candidate genes related to skeletal muscle aging and prospective therapeutic targets for effective treatments. The age-line-related genes (ALRGs) and age-line-related transcripts (ALRTs) were investigated using the gene expression profiles of GSE47881 and GSE118825 from the Gene Expression Omnibus (GEO) database. The protein-protein interaction (PPI) networks were performed to identify the key molecules with Cytoscape, and Gene Set Enrichment Analysis (GSEA) was used to clarify the potential molecular functions. Two hub molecules were finally obtained and verified with quantitative real-time PCR (qRT-PCR). The results showed that the expression of mitochondria genes involved in mitochondrial electron transport, complex assembly of the respiratory chain, tricarboxylic acid cycle, oxidative phosphorylation, and ATP synthesis were down-regulated in skeletal muscle with aging. We further identified a primary hub gene of CYCS (Cytochrome C) and a key transcription factor of ESRRA (Estrogen-related Receptor Alpha) to be associated closely with skeletal muscle aging. PCR analysis confirmed the expressions of CYCS and ESRRA in gastrocnemius muscles of mice of different ages were significantly different, and decreased gradually with age. In conclusion, the main cause of skeletal muscle aging may be the systematically reduced expression of mitochondrial functional genes. The CYCS and ESRRA may play significant roles in the progression of skeletal muscle aging and serve as potential biomarkers for future diagnosis and treatment.

## Graphical Abstract

**Figure:**
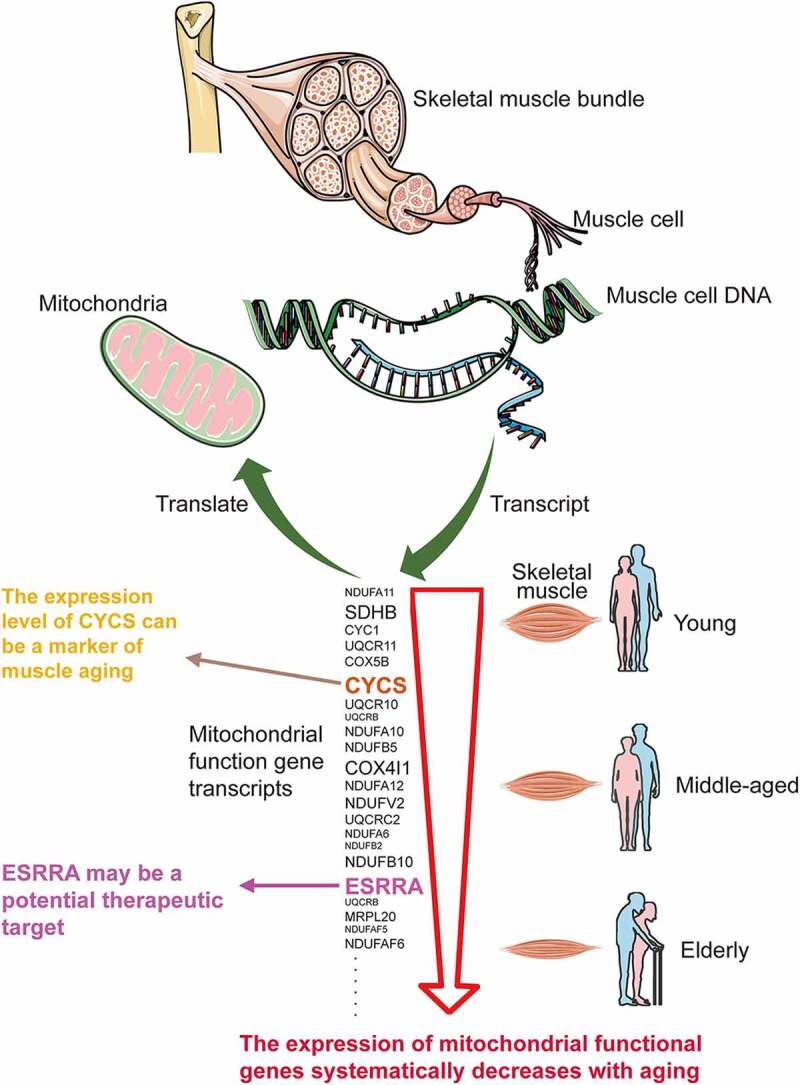

## Introduction

Age-related sarcopenia is a major public health priority in a world, where the aging population has become a dominant global trend. According to the China Development Research Foundation (CDRF: https://www.cdrf.org.cn), the elderly aged ≥65 years are projected to account for 27.9% of the Chinese population by 2050. Sarcopenia is a progressive and generalized skeletal muscle damage with advancing age, characterized by a reduction in skeletal muscle fiber, an increase in visceral fat and a disruption in muscle metabolism [1]. Skeletal muscle is the most abundant tissue constituting around half of the human body, and it is thus essential for physical function and health. Age-related decrease of muscle mass and strength could lead to lower quality of life and higher risk of adverse outcomes, including falls, frailty, disability, and mortality [2–4]. Sarcopenia may be related to the imbalance of protein synthesis and degradation, the failure of muscle stem cells, the aging of muscle cells, and the dysfunction of mitochondria, all of which may be ascribed to altered gene expression levels during skeletal muscle aging [5,6].

Previously, several studies focused on comparing the differences in skeletal muscle gene expression profiles between young and elderly individuals [7–11], but they ignored the linear expression patterns that occur as individuals age. However, there were a large number of candidate genes in each of these studies, but no consistent result has been reached, and specific markers remain unknown. These above results may be interfered by possible confounding factors, which were insufficient to reflect the direct effect of aging on skeletal muscle. Bioinformatics is a powerful tool for identifying hub genes in various diseases [12–14], therefore it was necessary to give a comprehensive analysis of the mechanism and biomarkers related to skeletal muscle aging, as well as the use of the linear analysis method.

The novelty of this study was to explore the key molecules from age-line-related genes (ALRGs) in skeletal muscle by bioinformatic analyses, further identify potential markers for diagnosis and therapy of skeletal muscle aging. We firstly screened linear expression genes significantly associated with age-related changes in sarcopenia based on Gene Expression Omnibus (GEO) database, which were then further evaluated by Gene Set Enrichment Analysis (GSEA), pathway analysis and protein-protein interaction (PPI) network. Finally, we discovered that vast mitochondrial function genes were systematically down-regulated with aging, and CYCS and ESRRA were identified as potential biomarkers and therapeutic targets in skeletal muscle aging. These findings may contribute to better understanding the molecular mechanism in the development of skeletal muscle aging, and provide new insight for future studies.

## Materials and methods

### Gene expression profile data

Gene expression data series matrix files (GSE47881 and GSE118825) were downloaded and adopted from the GEO database (https://www.ncbi.nlm.nih.gov/geo) [15]. A total of 89 samples in GSE47881 had skeletal muscle gene expression data of people at different ages, 54 samples in GSE118825 had rat skeletal muscle gene expression data. The two datasets met the following criteria: (1) Human or animal skeletal muscle gene expression data; (2) The age span was relatively wider, and the span must cover youth to old age; (3) The age distribution density was relatively uniform, including the young, middle and the old age group; (4) Total sample size was not less than 30. In addition, the microarray data of the human skeletal muscle biopsy (GSE25941, GSE28392, GSE28422, GSE362, GSE38718, GSE5086, GSE674, GSE9013, GSE4667) were acquired from the GEO database, consisting of young (<30 years) and old (>60 years) groups.

### Identification of age-line-related genes (ALRGs)

All microarray gene expression signal values were pre-processed by the Affymetrix Microarray Suite 5.0 algorithm (MAS5). The relationships between ages and gene expressions in the GSE47881 and GSE118825 datasets were performed by Pearson correlation analysis and linear analysis. The transcript data which was conformed to normal distribution were used to calculate the correlation coefficient (r) and slope (b) of the linear equation between the values of gene expressions and ages. Microarray dataset with an absolute value of r ≥ 0.7, b ≥ 16 and *P* < 0.05 in GSE47881 was considered statistically significant, as well as an absolute value of r ≥ 0.9, b ≥ 12 and *P* < 0.05 in GSE118825. There were 400–500 transcripts with significant linear patterns between gene expression levels and ages, which were named age-line-related transcripts (ALRTs), and the corresponding genes named age-line-related genes (ALRGs).

### PPI network construction and Gene Set Enrichment Analysis (GSEA)

The STRING database (https://string-db.org) [16] was applied to predict protein-protein interactions among the ALRGs, and which was then imported into Cytoscape 3.7 [17] for constructing the protein-protein interaction (PPI) network of ALRGs (using MCODE plug: network scoring: Degree cutoff = 2, Cluster Finding: Node score cutoff = 0.2, K-core = 2, Max. depth = 100). Moreover, we performed module analysis to identify hub clustering modules in the PPI network by using Molecular Complex Detection (MCODE) [18]. Gene Set Enrichment Analysis (GSEA) was conducted to elucidate the potential biological processes, molecular functions, cellular components, and signal pathways of ALRGs via the ClueGo+Cluepedia plugin of Cytoscape software [19,20].

### Verification of Hub gene and Transcription Factor

The hub genes of ALRGs in the dataset GSE47881 were screened out by Cytohubba plugin with 9 topological analysis methods including Degree, Edge Percolated Component (EPC), Maximum Neighborhood Component (MNC) and centralities based on shortest paths, such as Bottleneck (BN), EcCentricity, Closeness, Radiality, Betweenness, and Stress [21]. Based on the top 20 genes scored by each algorithm, the intersection was identified with the Ven diagram. Then we identified the differential expression of the hub gene between skeletal muscle samples of old and young in various datasets. The Iregulon app [22] in Cytoscape was used to predict the Transcription Factor in the PPI network, as well as the target genes based on the Signatures databases of Gene sets (MsigDB: http://software.broadinstitute.org/gsea/msigdb, GeneSigDB: https://genesigdb.org) [23,24]. The age-related significant changes of the Transcription Factor were investigated in skeletal muscle gene expression profiles, and the overall effect was calculated by Meta-analysis. The Co-expression profile of the Transcription Factor in NCBI (https://www.ncbi.nlm.nih.gov) was obtained from in Coexpedia (http://www.coexpedia.org) [25]. Moreover, we used KEGG pathway analysis to investigate the possible function of the Transcription Factor.

### RNA extraction and quantitative real-time PCR (qRT-PCR)

The study was approved by the Experimental Animal Ethics Committee of the First Affiliated Hospital of Nanjing Medical University (NO.2012013). All the experimental procedures were conducted according to the guidelines of the National Institutes of Health for the care and use of laboratory animals. Total RNA was isolated from gastrocnemius muscles from C57Bl/6 mice using Trizol reagent (Invitrogen, Carlsbad, USA) according to the manufacturer’s protocol. RNA was then reverse-transcribed to cDNA using the HiScript Synthesis kit (Vazyme, Nanjing, China). The qRT-PCR was performed using the StepOnePlus real-time PCR system (Applied Biosystems, CA, US) with the Fast SYBR Green Master Mix (Roche, Germany). The primers were synthesized by Sangon Biotech (Shanghai, China) as follows, CYCS (NCBI Gene ID 13,063: forward-5ʹ- CCA AAT CTC CAC GGT CTG TTC-3ʹ; reverse-5ʹ- ATC AGG GTA TCC TCT CCC CAG-3ʹ), ESRRA (NCBI Gene ID 26,379: forward-5ʹ- CTC AGC TCT CTA CCC AAA CGC-3ʹ; reverse-5ʹ- CCG CTT GGT GAT CTC ACA CTC-3ʹ), β-actin (forward-5ʹ- GGC TGT ATT CCC CTC CAT CG-3ʹ; reverse-5ʹ- CCA GTT GGT AAC AAT GCC ATG T-3ʹ). Relative gene expression levels were calculated with the formula of 2-ΔΔCT.

### Statistical analysis

The non-normal distribution data were excluded in this study, we retained normal distribution data for subsequent analysis. Pearson correlation test was conducted to analyze the relationship between gene expression levels and ages. Gene expression data were expressed as mean ± standard deviation, and T-test was used to compare two groups, P < 0.05 was considered statistically significant. Statistical tests were performed on R version 3.4.3 software, and Meta-analysis was used by Review Manager 5.3 software.

## Results

In this work, we used bioinformatics analysis to identify the hub genes from ALRTs in skeletal muscle aging based on the GEO database, and explore potential therapeutic targets. The expressions of vast mitochondrial function genes were found to be correlated with skeletal muscle aging, and to be systematically down-regulated with age. Finally, we discovered a hub gene CYCS and a transcription factor ESRRA may play important roles in the progression of skeletal muscle aging, and we further verified their expression levels in skeletal muscle of mice of different ages decrease with aging by qRT-PCR.

### Age-line-related genes (ALRGs)

A total of 416 ALRTs and the corresponding 374 ALRGs were screened in the GSE47881 dataset from biopsy samples of human Vastus lateralis muscle based on GEO database using Affymetrix Microarray Suite 5.0 algorithm (MAS5), Another 458 ALRTs and the corresponding 418 ALRGs were found in GSE118825 dataset from rat gastrocnemius muscle samples.

### PPI networks of ALRGs in skeletal muscle

**Figure 1.**
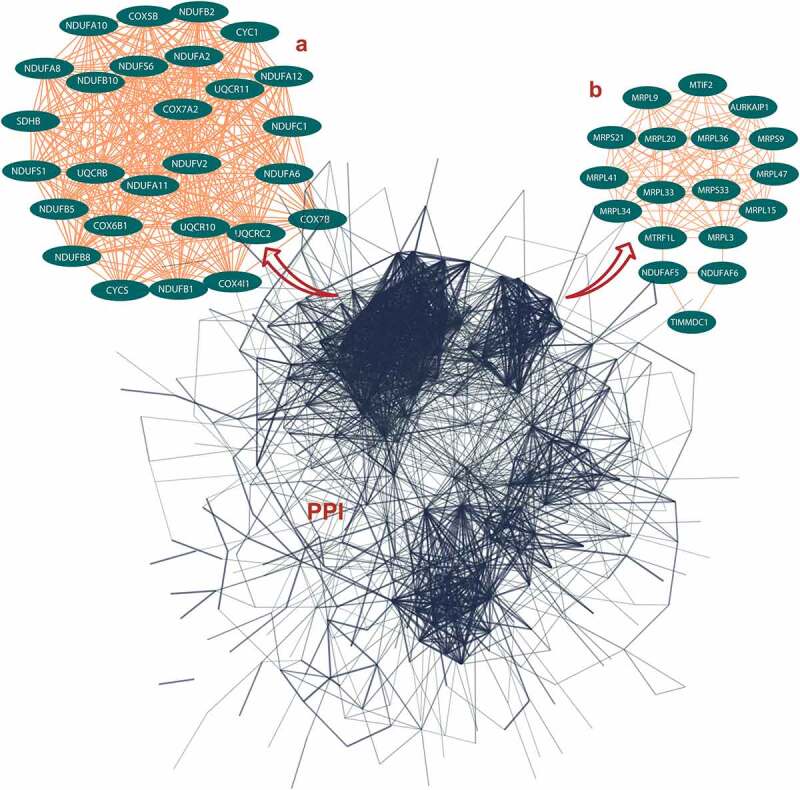
Protein-protein interaction (PPI) network construction of age-line-related genes (ALRGs) in GSE47881 dataset by the STRING database (https://string-db.org/). (a) The first Cluster proteins (MCODE Cluster score = 30.8) and (b) the second cluster proteins (MCODE Cluster score = 15.6) associated with mitochondrial function

**Figure 2.**
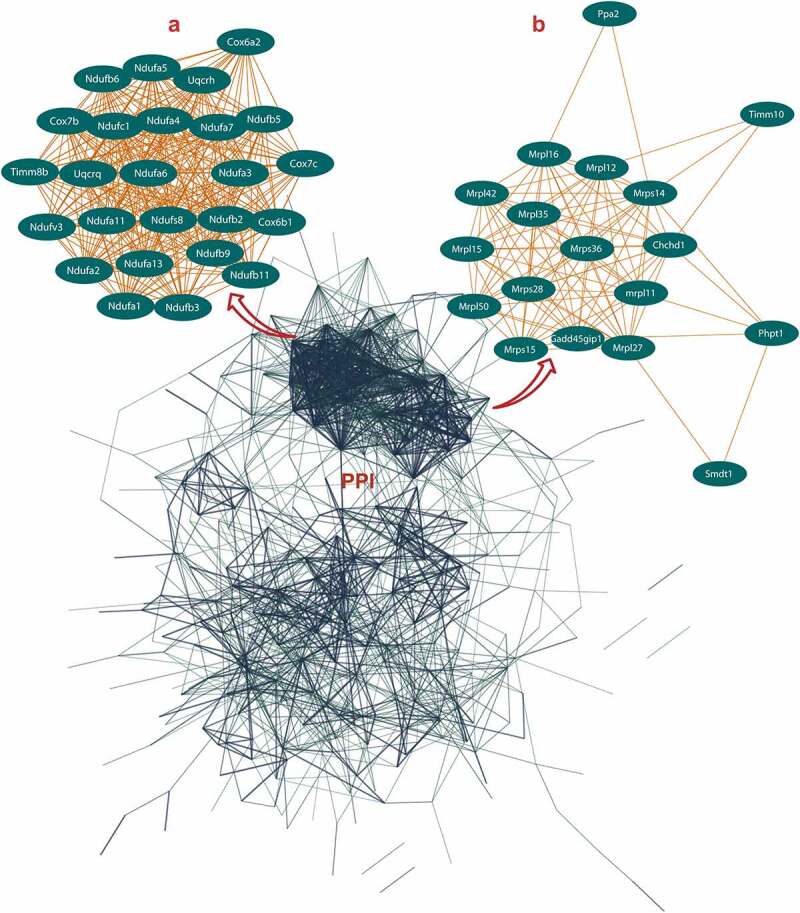
Protein-protein interaction (PPI) network construction of age-line-related genes (ALRGs) in GSE118825 dataset by the STRING database (https://string-db.org/). (a) The first Cluster proteins (MCODE Cluster score = 24.9) and (b) the second cluster proteins (MCODE Cluster score = 12) associated with mitochondrial function

**Figure 3.**
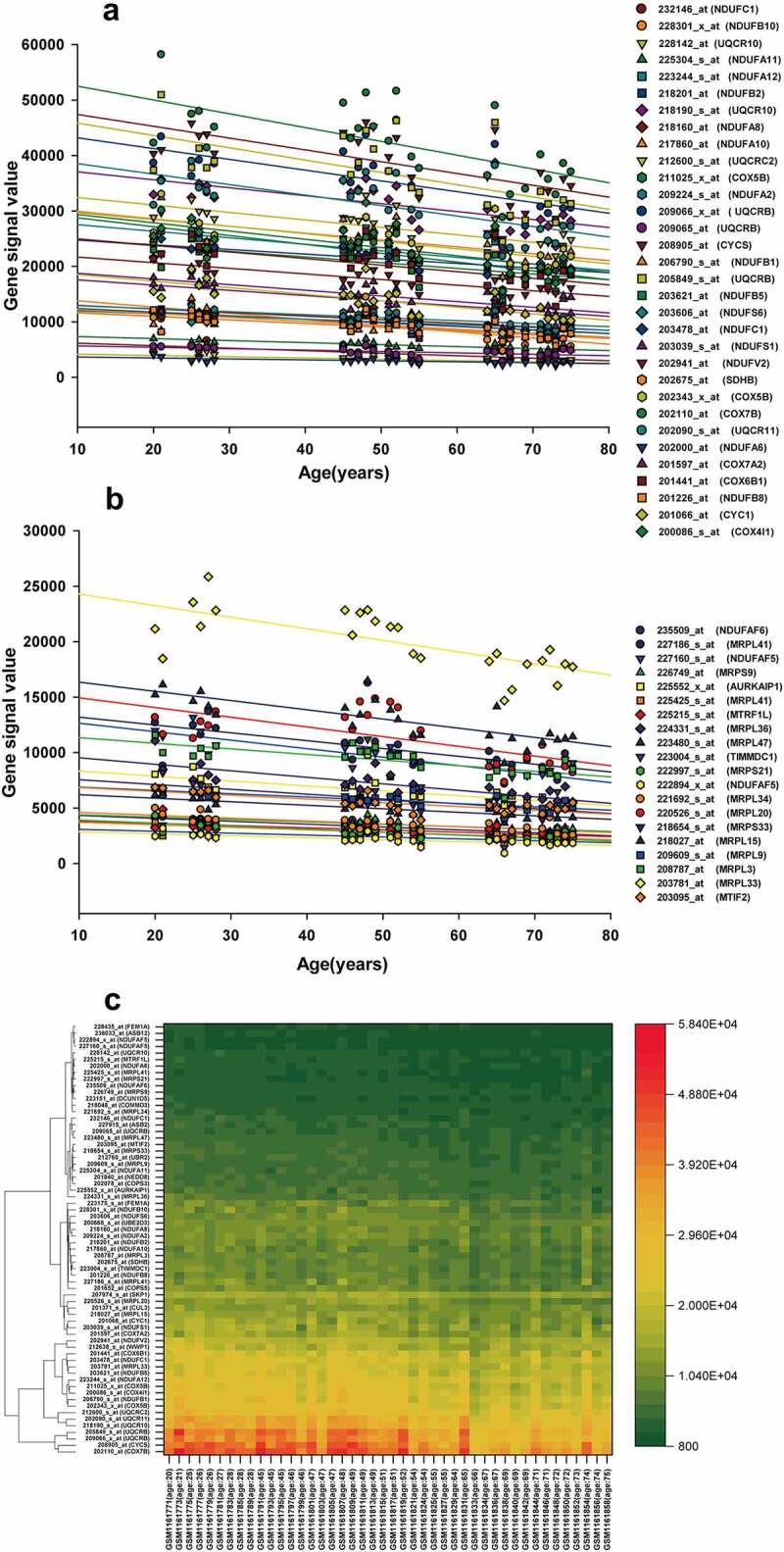
(a, b) Genes corresponding to the cluster proteins A and B in GSE47881 dataset decreased with age. (c) Heat map of the cluster genes in GSE47881 dataset

**Figure 4.**
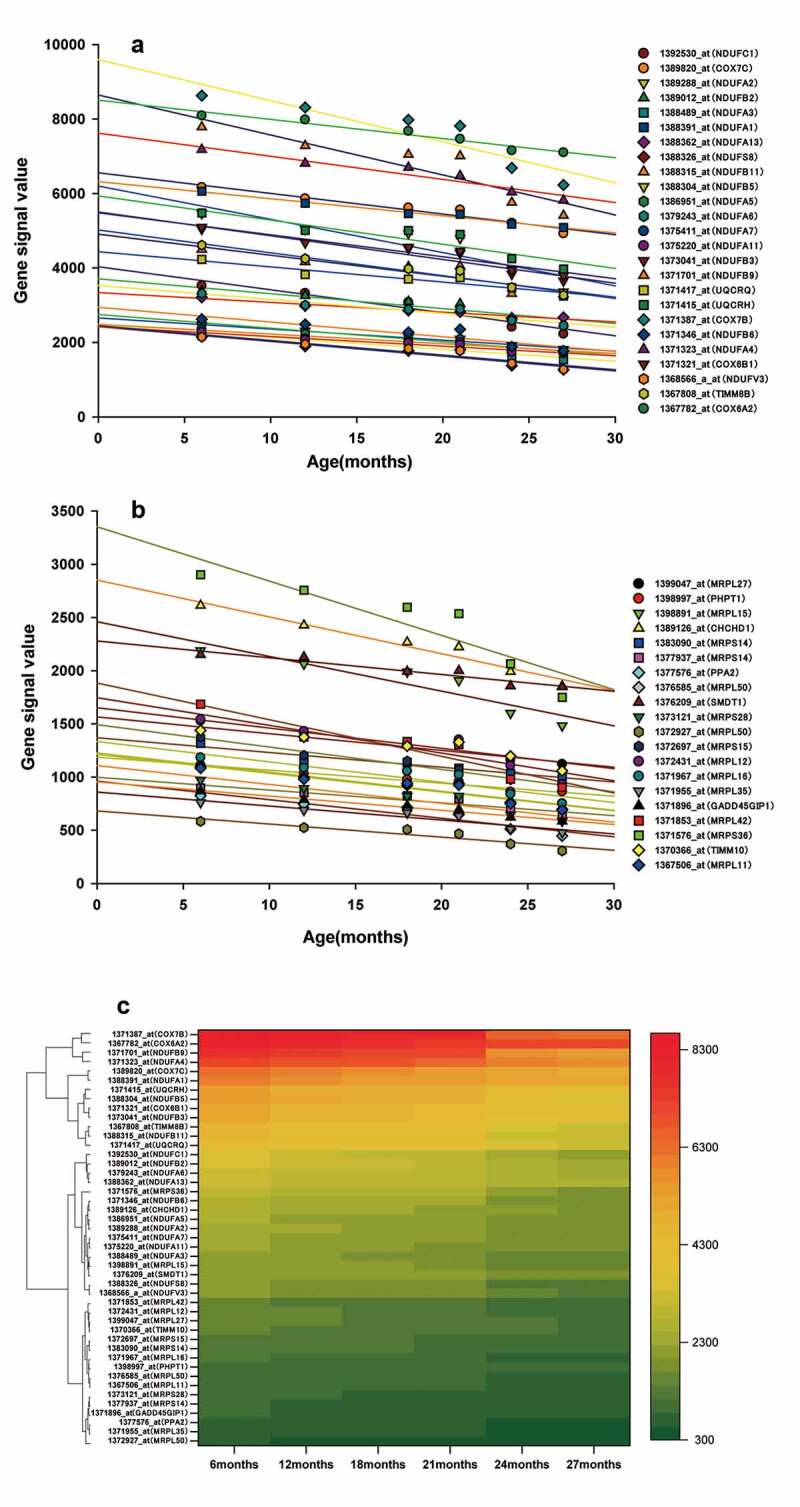
(a, b) Genes corresponding to the cluster proteins A and B in GSE118825 dataset decreased with age. (c) Heat map of the cluster genes in GSE118825 dataset

The PPI networks based on ALRGs were established using the STRING online tool. The core region of the PPI network in both Humans or Rats is mitochondria function proteins, including mitochondrial membrane proteins and respiratory chain proteins. A total of two hub clusters from GSE47881 were selected by the scores computed in MCODE. The first cluster (MCODE Cluster score = 30.8) proteins included NDUFA11, SDHB, CYC1, UQCR11, NDUFB1, COX6B1, COX7B, NDUFS1, COX5B, CYCS, UQCR10, UQCRB, NDUFA10, NDUFB5, COX4I1, NDUFA12, NDUFV2, UQCRC2, NDUFA6, NDUFB2, NDUFC1, NDUFB8, NDUFA2, NDUFB10, COX7A2, NDUFS6 and NDUFA8 (Figure 1a). The second cluster (MCODE Cluster score = 15.6) proteins included MRPL15, MRPL20, NDUFAF5, MRPL34, MRPL41, MRPL47, NDUFAF6, MRPS9, MTRF1L, MTIF2, MRPS33, TIMMDC1, MRPL36, AURKAIP1, MRPL33, MRPS21, MRPL3 and MRPL9 (Figure 1b). Another two hub-clusters of PPI network from the GSE118825 dataset were generated, the first cluster (MCODE Cluster score = 24.9) included NDUFA6, NDUFA7, UQCRH, NDUFB9, NDUFA13, NDUFA11, NDUFB11, NDUFB2, COX7B, NDUFA1, NDUFB5, COX7C, NDUFA4, UQCRQ, TIMM8B, NDUFC1, NDUFB3, NDUFV3, COX6A2, NDUFS8, COX6B1, NDUFA2, NDUFB6, NDUFA3 and NDUFA5 (Figure 2a), and the second Cluster (MCODE Cluster score = 12) protein included SMDT1, MRPL11, MRPL15, GADD45GIP1, MRPL50, TIMM10, PHPT1, MRPS28, MRPL42, MRPS15, MRPL27, CHCHD1, MRPL35, PPA2, MRPS14, MRPL16, MRPL12 and MRPS36 (Figure 2b). The expression levels of mitochondrial-related genes decreased with aging in these two PPI networks, as shown in the regression line and the heatmap (Figures 3 and 4).

### Gene Set Enrichment Analysis (GSEA) of ALRGs

**Figure 5.**
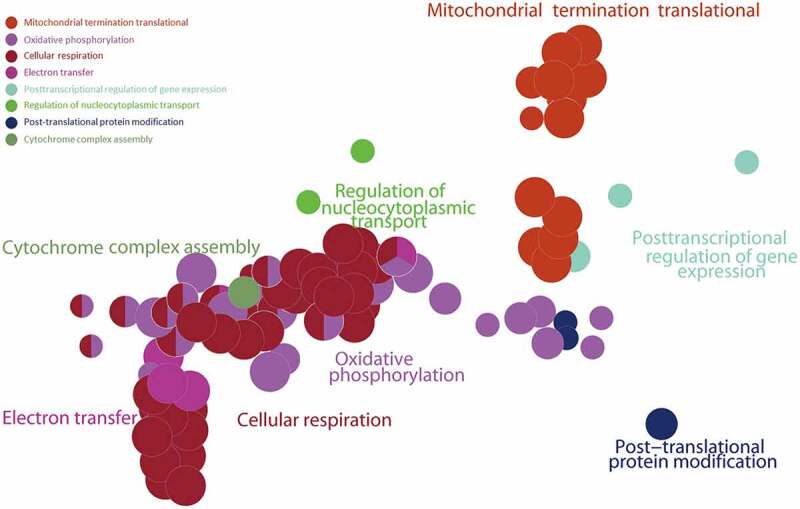
Gene Set Enrichment Analysis (GSEA) of the ALRGs in GSE47881 dataset links with mitochondrial biological processes like mitochondrial termination translation, oxidative phosphorylation, cellular respiratory, electron transfer. *Note*: Different colored balls represent different signaling pathways, different ball size represents the number of genes enriched in the pathway, and different colors in the same ball represent the same genes in two biological processes

**Figure 6.**
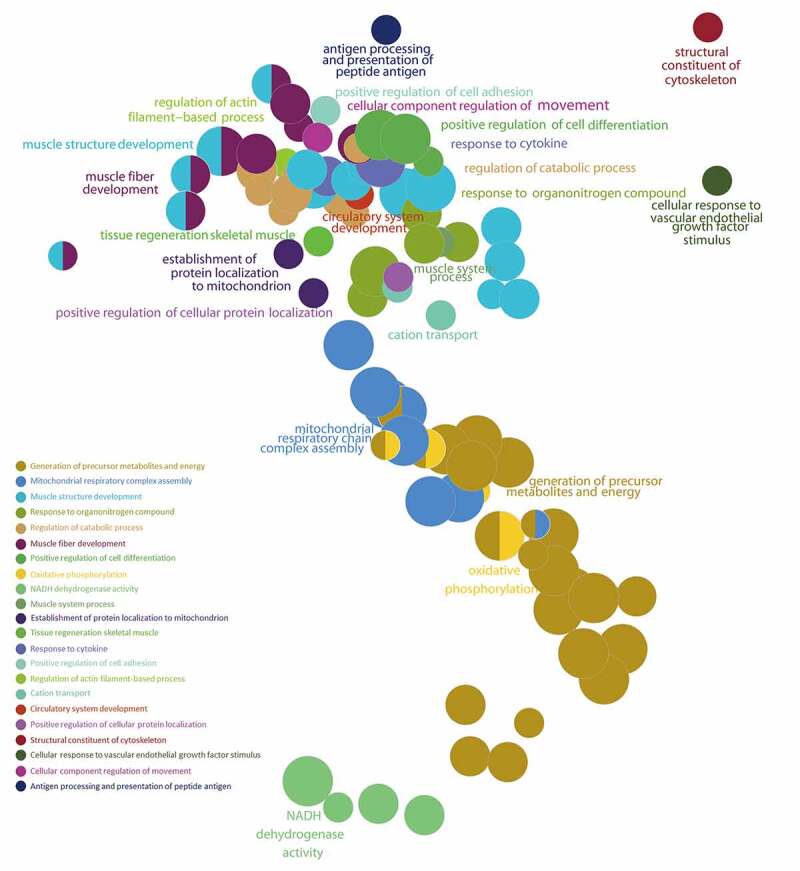
Gene Set Enrichment Analysis (GSEA) of the ALRGs in GSE118825 dataset links with mitochondrial biological processes like mitochondrial respiratory chain complex assembly, generation of precursor metabolites and energy, oxidative phosphorylation, NADH dehydrogenase activity. *Note*: Different colored balls represent different signaling pathways, different ball size represents the number of genes enriched in the pathway, and different colors in the same ball represent the same genes in in two biological processes

**Figure 7.**
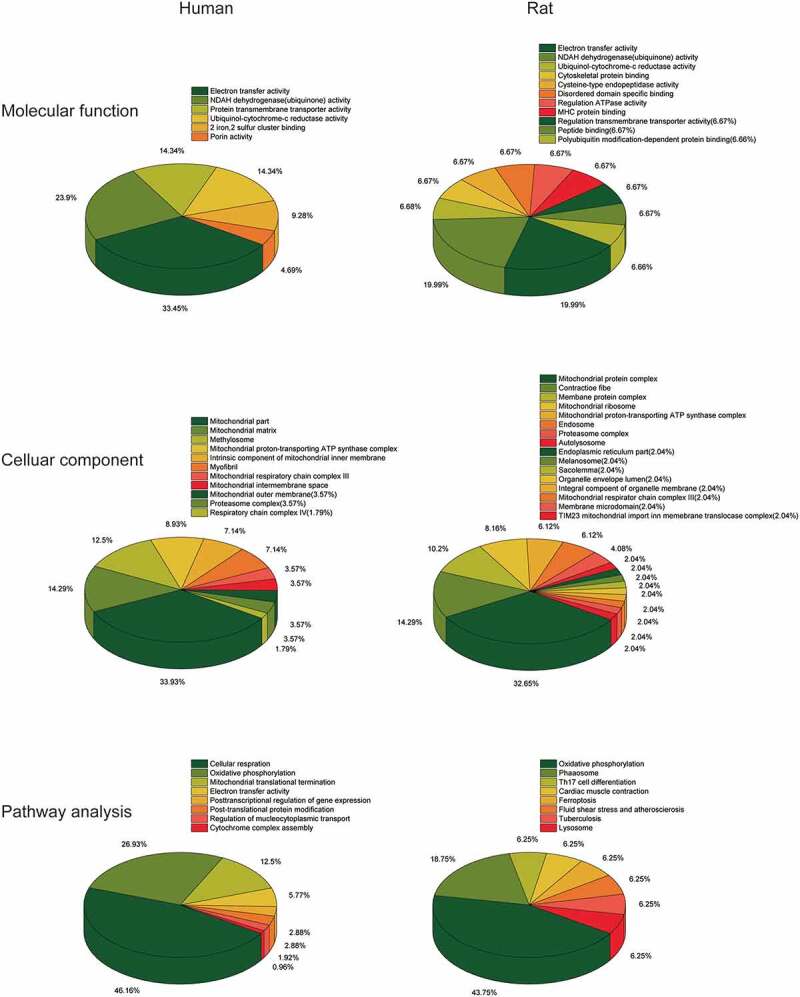
(a) Molecular Function gene sets of the ALRGs in GSE47881 and GSE118825 mainly involved electron transfer activity, NADH dehydrogenase activity, ubiquinol-cytochrome C reductase activity. (b) Celluar components largely involved the mitochondria complex. (c) Pathways analysis primarily involved cellular respiration, oxidative phosphorylation, and electron transport

The GSEA results showed that the ALRGs expressions were enriched in the mitochondria, which refer to a variety of biological processes such as electron transport chain, cellular respiration, Tricarboxylic acid (TCA) cycle, oxidative phosphorylation, and cytochrome assembly (Figures 5–6). Furthermore, the molecular functions of the corresponding proteins based on ALRGs focus on electron transfer activity, NADH dehydrogenase activity, and ubiquinol-cytochrome c-reductase activity, which were carried out primarily in mitochondria. No matter in humans and rats, pathways are enriched in oxidative phosphorylation in mitochondria (Figure 7).

### The hub gene of ALRGs

**Figure 8.**
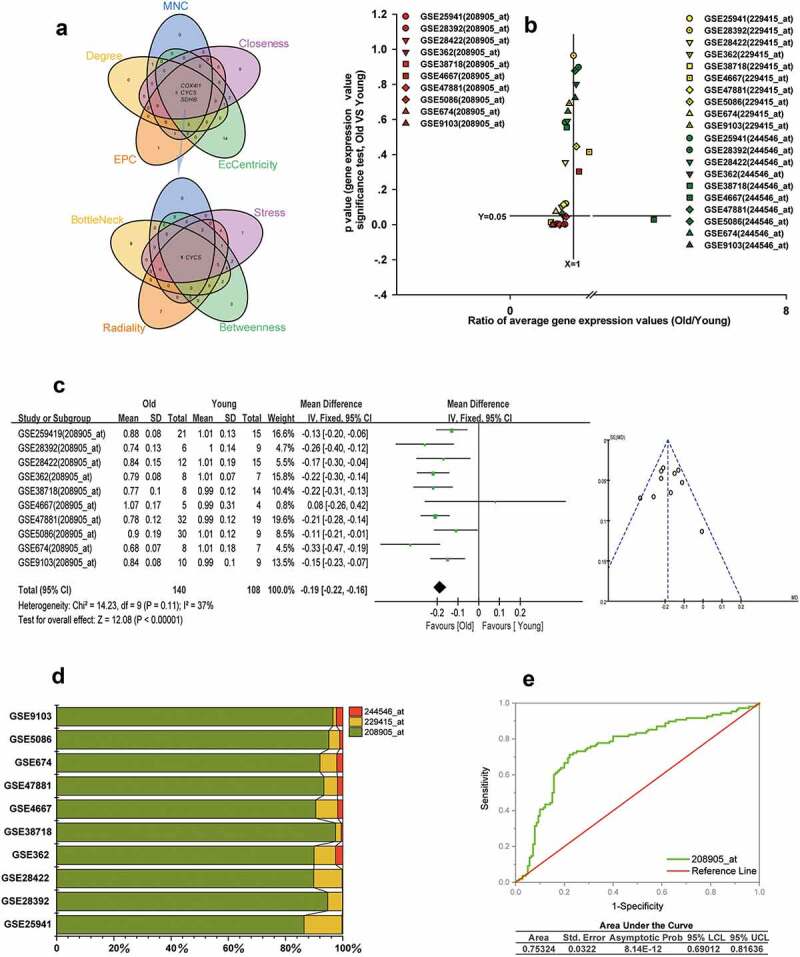
CYCS is the hub gene of ALRGs in human skeletal muscle. (a) The candidate hub gene of ALRGs in GSE47881 dataset by nine topological algorithms from CytoHubba of Cytoscape software. (b) A scatter plot between old/young ratio of average relative expression of CYCS (208905_at, 229415_at and 244546_at transcripts) and P-value significance in different GSE skeletal muscle datasets. (c) Meta-analysis of multiple studies on the difference of expression values of 208905_at transcript in skeletal muscle between old and young people. (d) The 208905_at transcript expression takes the largest proportion of CYCS transcripts in each GSE skeletal muscle dataset. (e) ROC curves for relative values of 208905_at expression between old and young people based on the GSE skeletal muscle datasets

The hub gene CYCS was obtained from the intersection of the top 20 genes scored by each of the nine topological analyses, as shown in Figure 8a. The numbers in the Venn diagram represented the intersection set of hub genes obtained from topological analyses. There were three gene transcripts for CYCS in Affymetrix GeneChip, 208905_at, 229415_at, and 244546_at, respectively. We found that the expression of 208905_at was significantly lower in the elderly than in young people in GSE25941, GSE28392, GSE28422, GSE362, GSE38718, GSE5086, GSE674, GSE9013 and GSE47881 datasets (p < 0.05), but not in GSE4667 (p > 0.05). However, neither the 229415_at expression nor the 244546_at expression differed significantly between the two groups in the above datasets (p > 0.05) (Figure 8b). We also conducted a meta-analysis based on the two groups to calculate the value of the 208905_at expression in these cohorts from the GEO dataset. Our results indicated that old people showed a decreased 208905_at expression in skeletal muscle compared to young people (p < 0.00001). (Figure 8c). Moreover, the signal value of 208905_at was significantly higher than that of 229415_at, 244546_at in the 10 cohorts. The 208905_at expression was responsible for most of these three transcripts in each dataset, accounting for at least 86%. These indicated that the 208905_at was the hub transcript of the CYCS in skeletal muscle (Figure 8d). We plot the receiver operating characteristic (ROC) curve, and found that the area under the curve (AUC) of 208905_at was 0.73, and the asymptotic probability was 8.14E-12 (Figure 8e).

### The transcriptional regulators of ALRGs

**Figure 9.**
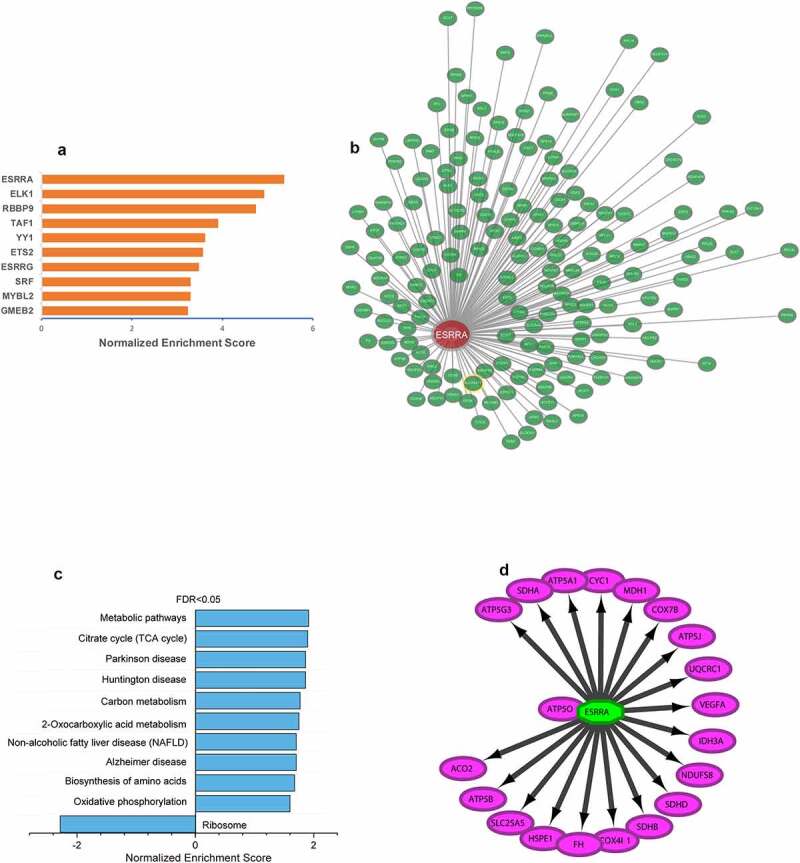
ESRRA is a key transcription factor of ALRGs in human skeletal muscle. (a) The potential transcription factors in the PPI network of ALRGs in GSE47881 dataset by the iRegulon plugin of Cytoscape. (b) The co-expression network of ESRRA by the Coexpedia internet tool (http://www.coexpedia.org). (c) Pathway enrichment analysis of the co-expression network. (d) The prediction of ESRRA target genes from the MSigDB and GeneSigDB databases

**Figure 10.**
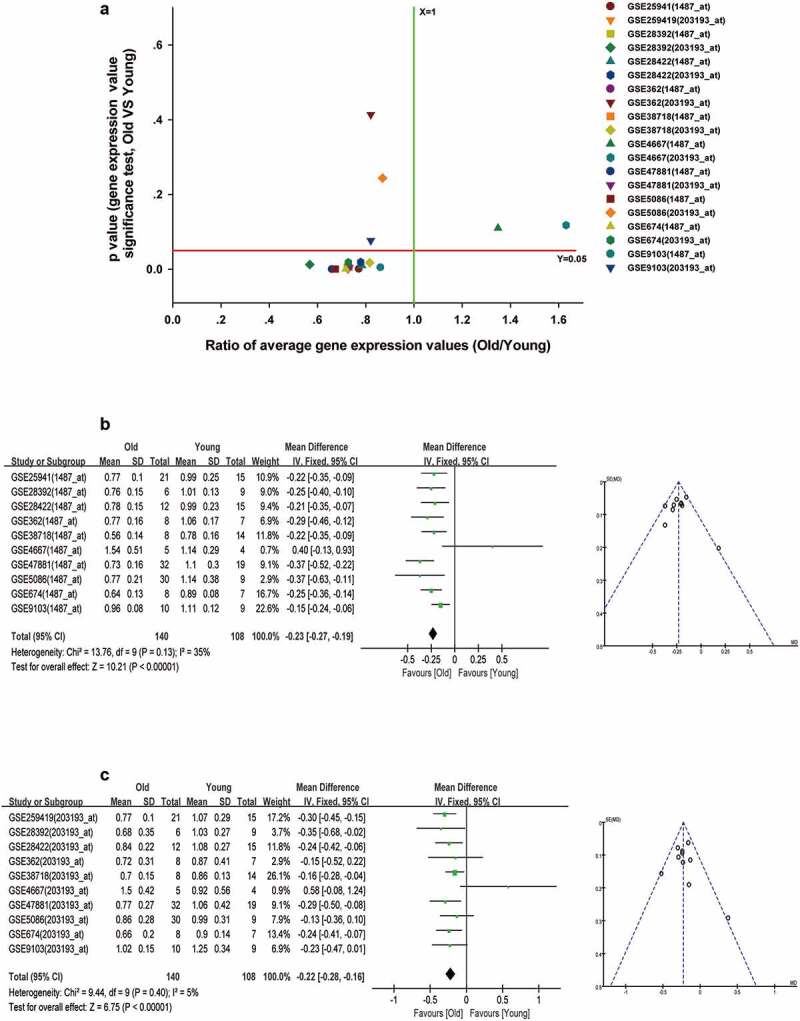
Decreased expression of ESRRA in skeletal muscle of elderly people. (a) A scatter plot between old/young ratio of average relative expression of ESRRA (1487_at and 203193_at transcripts) and P-value significance in different GSE skeletal muscle datasets. (b-c) Meta-analysis of multiple studies on the difference of expression values of 1487_at (b) and 203193_at (c) transcript in skeletal muscle between old and young people

We used the Iregulon app in Cytoscape software with default settings to predict transcription factors for mitochondria function in the PPI networks of ALRGs of the GSE47881 dataset. The results showed that ESRRA got a normalized enrichment score (NES) of 5.358, which was higher than other transcription factors (Figure 9a). we constructed an ESRRA co-expression network using microarray data from the GEO database on http://www.coexpedia.org (Figure 9b). The result of pathway analysis revealed that these proteins were mostly enriched in metabolic pathways related to mitochondrial function, such as the citrate cycle (TCA cycle), carbon metabolism, 2-Oxocarboxylic acid metabolism, Oxidative phosphorylation (Figure 9c). A total of 20 target genes of ESRRA were identified with the iRegulon app for Cytoscape based on the Molecular Signatures Database (MSigDB) and Gene Signature DataBase (GeneSigDB), 19 of which are mitochondrial function-associated genes (Figure 9d). We also explored the expression of ESRRA in the elderly and young people in published studies, and found that most of the two transcripts of ESRRA in old people were significantly down-regulated compared to young people (Old/Young < 1, *P* < 0.05, Figure 10a). The results were further verified by a Meta-analysis of studies on the 1487_at and 203193_at expression (Figure 10(b,c)).

### The CYCS and ESRRA expressions by quantitative real-time PCR (qRT-PCR)

**Figure 11.**
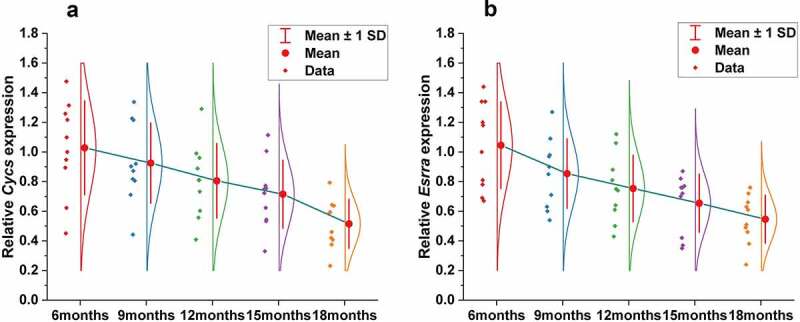
Decreased expression of CYCS and ESRRA in skeletal muscle with aging by qRT-PCR. Gene expression of CYCS (a) and ESRRA (b) in mice is significantly different across ages (6 months, 9 months, 12 months, 15 months and 18 months), and decreased gradually with aging

To validate the results obtained from bioinformatics analysis, we detected CYCS and ESRRA mRNA expressions in the gastrocnemius muscles of mice of different ages (6 months, 9 months, 12 months, 15 months and 18 months). The results showed that either CYCS or ESRRA expression was significantly different among groups (P < 0.001), and decreased gradually with age (Figure 11).

## Discussion

In 1997, Rosenberg posed the issue of whether age-related changes in the structure and function of skeletal muscles were a physical disease or a process of normative aging [26], but no definitive answer has yet been found. It is worth mentioning that skeletal muscle loss has a significant impact on elderly people’s quality of life, but the potential mechanisms need to be further explored. Our aim was to identify age-line-related genes in skeletal muscle aging based on public microarray data, and further explore the possible mechanisms.

In the present study, we have demonstrated that the hub genes of ALRGs were identified as mitochondrial function-associated genes in the muscle of humans and rats, and their expression levels are down-regulated with aging, indicating that skeletal muscle loss with age in vivo may attribute to the decreased mitochondrial content and quality. However, there are conflicting views on mitochondria and skeletal muscle aging. Previous studies have suggested that the long-term accumulation of reactive oxygen species (ROS) may cause damage to mitochondrial structure and function in skeletal muscle, and that subsequently damaged mitochondria generate more ROS [27–30], but this viewpoint is changing [31–34]. Conley KE et al. [35]found that the mitochondrial oxidative capacity per unit of muscle in elderly people was nearly 50% lower than that in adult subjects, which may ascribe to the reduced mitochondrial content with aging. Researchers were increasingly focusing on focused on mitochondria energy metabolism disorder and calcium homeostasis imbalance [36–42]. Our study revealed that the hub genes of ALRGs in skeletal muscle associated with mitochondrial energy metabolism. GSEA results showed that the enriched gene sets were strongly linked to mitochondria-related pathways such as the electron transport chain, cellular respiration, TCA cycle (or citric acid cycle), oxidative phosphorylation, and cytochrome complex assembly. These findings indicated that aging could alter the expression of mitochondrial energy metabolism-related genes, resulting in an energy metabolism disorder that eventually leads to skeletal muscle mass loss and function reduction.

More importantly, as a hub gene of ALRGs, CYCS expression levels were decreased in an age-dependent manner. Combined with the results of Meta-analysis and ROC curve, we speculate CYCS could be a useful biomarker for skeletal muscle aging. Of the three transcripts in CYCS, 208905_at transcript expression in skeletal muscle was significantly higher than the other two transcripts. CYCS is a central element of mitochondrial electron transport proteins that involved in ATP synthesis [43,44]. Once CYCS binds to Apaf-1, caspase-9 is activated to form a large caspase-activating complex known as apoptosome [45]. Jarr KU et al. [46]reported that CYCS expression was decreased with age-related cardiac dysfunction, suggesting that CYCS was an important determinant of muscle function. In our study, CYCS was selected as the representative gene of mitochondrial, which has an important effect on mitochondrial energy metabolism. We also demonstrated that the expression levels of CYCS decreased significantly with aging, which was not previously reported. It is therefore of particular clinical significance to detect the expression of CYCS in skeletal muscle for elderly people.

With the decreased expression levels of genes related to mitochondria function, we examined the transcription factors involved in mitochondrial biogenesis. We discovered that the ESRRA expression was significantly reduced in aged skeletal muscle, and among the 20 predicted target genes of ESRRA, 19 genes were found to be associated with mitochondrial function. Therefore, ESRRA was considered as the major transcription factor affecting mitochondrial energy metabolism in skeletal muscle during aging. ESRRA is a nuclear steroid hormone receptor, which could stimulate the expression of pyruvate dehydrogenase kinase isoform 4 gene and inhibit the transcription of phosphoenolpyruvate carboxykinase gene [47,48]. ESRRA is associated with most of the gene regulatory regions that encode enzymes involved in the TCA cycle, including *Aco2, Idh3a, Idh3b, Sdha, Sdhb, Sdhc, Sdhd, Ogdh, Cs and Sdhd*. Moreover, ESRRA is responsible for modulating the expression of more than 100 genes involved in the mitochondrial electron transport chain, including NADH dehydrogenase complex, ubiquinone-cytochrome C reductase, several cytochrome c oxidase subunits, and the ATPase superfamily [49,50]. ESRRA is thought to be engaged in mitochondrial energy metabolism as a key transcription factor, which plays an important role in ATP synthesis during aerobic respiration [51–56]. Given the specific characteristic of ESRRA in mitochondrial function, it could be a potential therapeutic target for elderly patients with sarcopenia.

There were also several limitations in this study. the data of our study were collected from public databases, but the quality of data could hardly be guaranteed, which might lead to inaccurate results. Second, we were unable to perform subgroup analyses by gender, due to the lack of gender information on samples in GSE47881. Considering that skeletal muscle mass and sex hormones differ between male and female, it is necessary to collect more clinical samples in subsequent experimental studies to explore the gender-specific differences in skeletal muscle aging. Third, although the CYCS and ESRRA were identified as biomarkers for sarcopenia, no functional experiments in vivo and vitro were conducted.

## Conclusion

In conclusion, this was the first study to investigate the age-liner-related genes in skeletal muscle aging based on bioinformatics and linear analysis. We found that vast mitochondrial gene expressions were down-regulated with aging, with CYCS as an important hub gene and ESRRA as a key transcription factor, both of which have the potential to be effective treatments in skeletal muscle aging. The present study could serve as a fundamental resource for understanding skeletal muscle aging, further studies are warranted to validate the clinical value of CYCS and ESRRA in sarcopenia patients.

## Data Availability

The datasets presented in this study can be found in the GEO database (https://www.ncbi.nlm.nih.gov/geo/).

## Highlights

(1) The expression of vast mitochondrial function genes in skeletal muscle was systematically down-regulated with aging.

(2) The hub gene CYCS and a transcription factor ESRRA were found to be associated closely with skeletal muscle aging.

(3) CYCS and ESRRA are expected to guide the diagnosis and treatment of skeletal muscle aging.

## Abbreviations

GEO: Gene Expression Omnibus database; ALRGs: Age-line-related genes;

ALRTs: Age-line-related transcripts; GSEA: Gene Set Enrichment Analysis; PPI: Protein-protein interaction; CYCS: Cytochrome c; ESRRA: Estrogen-related receptor alpha.
